# Topographic, tomographic, and corneal wavefront asymmetry in keratoconus: towards an eye asymmetry index EASIX

**DOI:** 10.1007/s00417-022-05642-5

**Published:** 2022-04-09

**Authors:** Juliane Mehlan, Johannes Steinberg, Vasyl Druchkiv, Toam Katz, Stephan Johannes Linke

**Affiliations:** 1 Department of Ophthalmology, University Medical Center Hamburg-Eppendorf (UKE), Martinistrasse 52, 20246 Hamburg, Germany; 2 CARE Vision/Clinica Baviera, Hamburg, Germany; 3 Zentrumsehstärke, Martinistr. 64, 20251 Hamburg, Germany

**Keywords:** Keratoconus—Intereye asymmetry—Anisometropia—Pentacam—Scheimpflug

## Abstract

**Purpose:**

The study aims to explore the intereye asymmetry in normal and keratoconic individuals and to evaluate the discriminant power of single and combined asymmetry parameters.

**Methods:**

This is a retrospective designed study including 414 patients who had Pentacam Scheimpflug topographic and tomographic imaging in both eyes: 124 subjects with bilateral normal corneas evaluated for refractive surgery and 290 with keratoconus. All elevation-, pachymetric-, and volumetric-based data (56 parameters) were electronically retrieved and analyzed. Intereye asymmetry was determined by subtracting the lowest value from the highest value for each variable. The degree of asymmetry between each subject’s eyes was calculated with intraclass correlation coefficients for all the parameters. Receiver operating characteristic curve was used to determine predictive accuracy and to identify optimal cutoffs of these values and combinations thereof.

**Results:**

In the normal/keratoconus subjects the median intereye asymmetries were 0.30/3.45 for K2 (flat) meridian, 0.03/0.25 for BFS front, 1.00/15.00 for elevation back BFS apex, and 7.00/29.00 for pachy min.

**Conclusions:**

In addition to Rabinowitz’s *K*_max_ intereye asymmetry we propose pachymetric, elevation-based, and high-order corneal wavefront intereye asymmetry parameters to improve the diagnostic armamentarium of keratoconus.


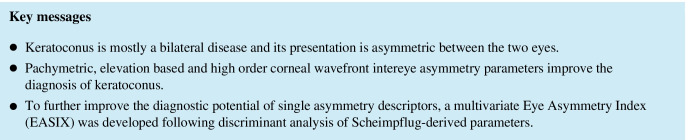


## Introduction

Although a high degree of symmetry was reported between fellow eyes in normal subjects [1–3], it has been reported many years ago that keratoconus is a bilateral non-inflammatory corneal ectasia [4] often presenting with an asymmetric intraindividual profile [3, 5–8]. Previous studies suggest that most patients have bilateral disease, and its presentation is asymmetric between the 2 eyes [7, 8]. Fifteen years ago, Rabinowitz and MacDonnell were the first to establish criteria for keratoconus that included a difference between the right and left central cornea power [3]. Zadnik et al. were among the first to systematically analyze the degree of asymmetry at presentation regarding corneal curvature, visual acuity, and corneal scarring and concluded that keratoconus is a markedly asymmetric, predominantly bilateral eye disease.

For the progression analysis, Shajari et al. suggest using *D*-index and KPI [9].

Today, the use of tomographic maps including pachymetric and topographic maps is almost mandatory in the clinical evaluation of refractive candidates and keratoconus patients [10]. Despite the high degree of asymmetry in keratoconus patients, the individual topographic and tomographic parameters of each cornea in potential candidates for refractive surgery are traditionally evaluated independently.

Compared with other ocular parameters such as intraocular pressure or cup-to-disk ratio, where a certain degree of asymmetry is considered “abnormal” [11], no general accepted cutoff values exist to distinguish between normal and keratoconic state regarding topographic and tomographic corneal asymmetries. Therefore, our retrospective study aims to provide intraindividual Scheimpflug-derived corneal asymmetry values to differentiate between normal and diseased states.

## Materials and methods

### Study population and clinical measures

Due to measurement quality and previous corneal surgery (keratoplasty, CXL, ICRS) only 290 of the keratoconus patients which referred to the cornea service of the Department of Ophthalmology of University Medical Centre Hamburg Eppendorf between 2008 and 2012 were enrolled in the study. The cohort for normative values consisted of 124 individuals attending Care Vision refractive clinics in Hamburg between 04 and 08/2013 for treatment of ametropias. Most of the subjects were candidates to undergo refractive surgery with excimer laser, either LASIK or PRK. Subjects exceeding the range for laser vision correction were candidates for phakic intraocular surgery or clear lens extraction.

Patients with a history of contact lens wear, previous eye surgery, or corneal disease were excluded from the study. Measurements were performed with a rotating Scheimpflug imaging system (Pentacam**®**, Oculus Inc., Dudenhofen, Germany). The patients were instructed to keep both eyes open and fixate on the black target in the center of the blue fixation beam. After attaining perfect alignment, the instrument automatically took a single scan containing 25 Scheimpflug images within 2 s. Only scans that had an examination quality specification graded as “OK” were saved.

Slit lamp biomicroscopic examination and refractive findings had also been recorded at initial assessment. A diagnosis of keratoconus for each eye had been determined at the time of initial diagnosis by experienced clinicians using the combined results from slit lamp biomicroscopy examination, refraction, and corneal mapping if the Pentacam analysis (setting: best-fit sphere; float: 8 mm) demonstrated a posterior elevation of ≥ 20 µm and a locally corresponding elevation of the anterior surface of ≥ 15 µm and/or a locally corresponding TPCT of < 500 µm in at least one eye of the patient. These criteria are recommended by the Pentacam interpretation guideline and coincide with our clinical experience. In addition, a topographic keratoconus classification (TKC) ≥ 1 and a Belin/Ambrosio Enhanced Ectasia Display (BAD) of > 2.0 verified the keratoconus diagnosis in the affected eye. An individual was defined as keratoconus patient if at least one eye showed a keratoconic pattern.

The study protocol was conducted according to the tenets of the Declaration of Helsinki of the World Medical Association regarding scientific research on human subjects. Informed consent was obtained from the subjects after explanation of the nature and possible consequences of the study. The analysis of the data was approved by the local ethical committee.

### Statistical analysis

The intraeye absolute differences were calculated for each parameter of interest. To investigate absolute agreement between partner eyes within healthy and keratoconus eyes intraclass correlation and Wilcoxon signed-rank test were computed. To compare absolute differences between the healthy and keratoconus eyes non-parametric Mann–Whitney test was used. Non-parametric approach was chosen because of non-normally distributed data, especially in keratoconus eyes, where the distributions are highly skewed. ROC analysis was used to calculate classification power of each parameter. The optimal cutoff was computed by minimizing the absolute difference between sensitivity and specificity.

**Fig. 1:**
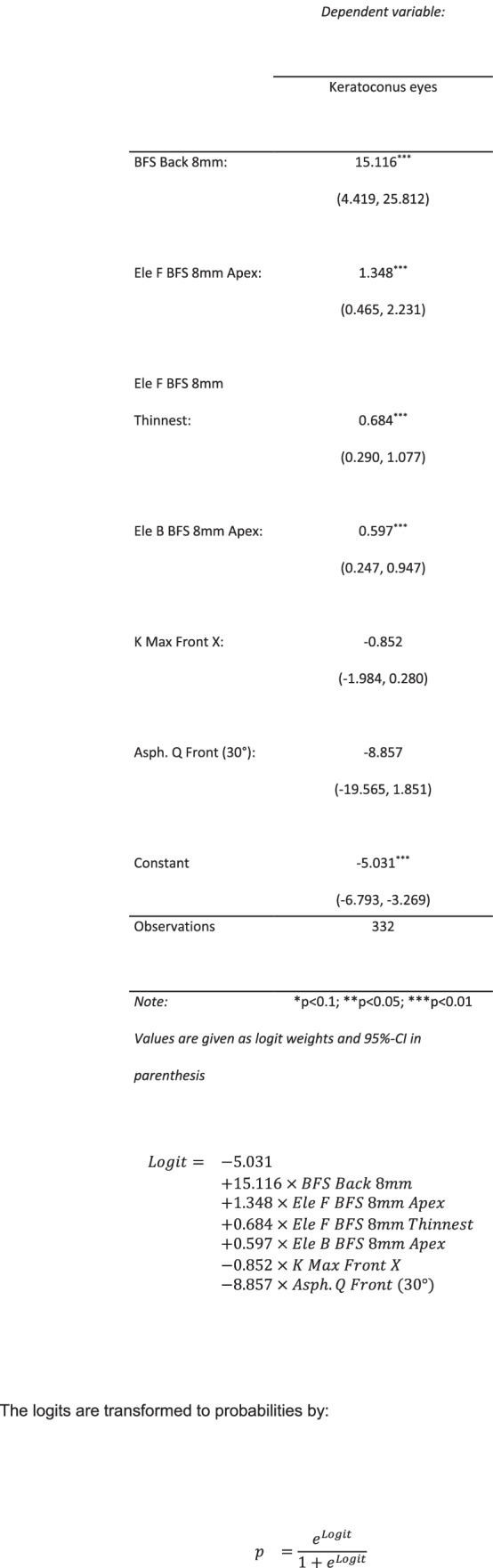
Logistic model and the estimated cut off point

Further multivariate analysis was performed. We divided the full data set in training and test datasets. Outcome-based partition was performed to preserve the overall class distribution in both data sets. For training data set 80% patients within each group were randomly selected resulting in 99 normal and 233 keratoconus patients in the training and 25 norm and 57 keratoconus patients in the test data sets. The initial set of variables was reduced by excluding multicollinear variables. The remaining variables in training data set were analyzed with logistic regression and reduced via backward selection algorithm based on Akaike information criterion (AIC). The final model and predictive equation is given in Fig. 1.

**Fig. 2:**
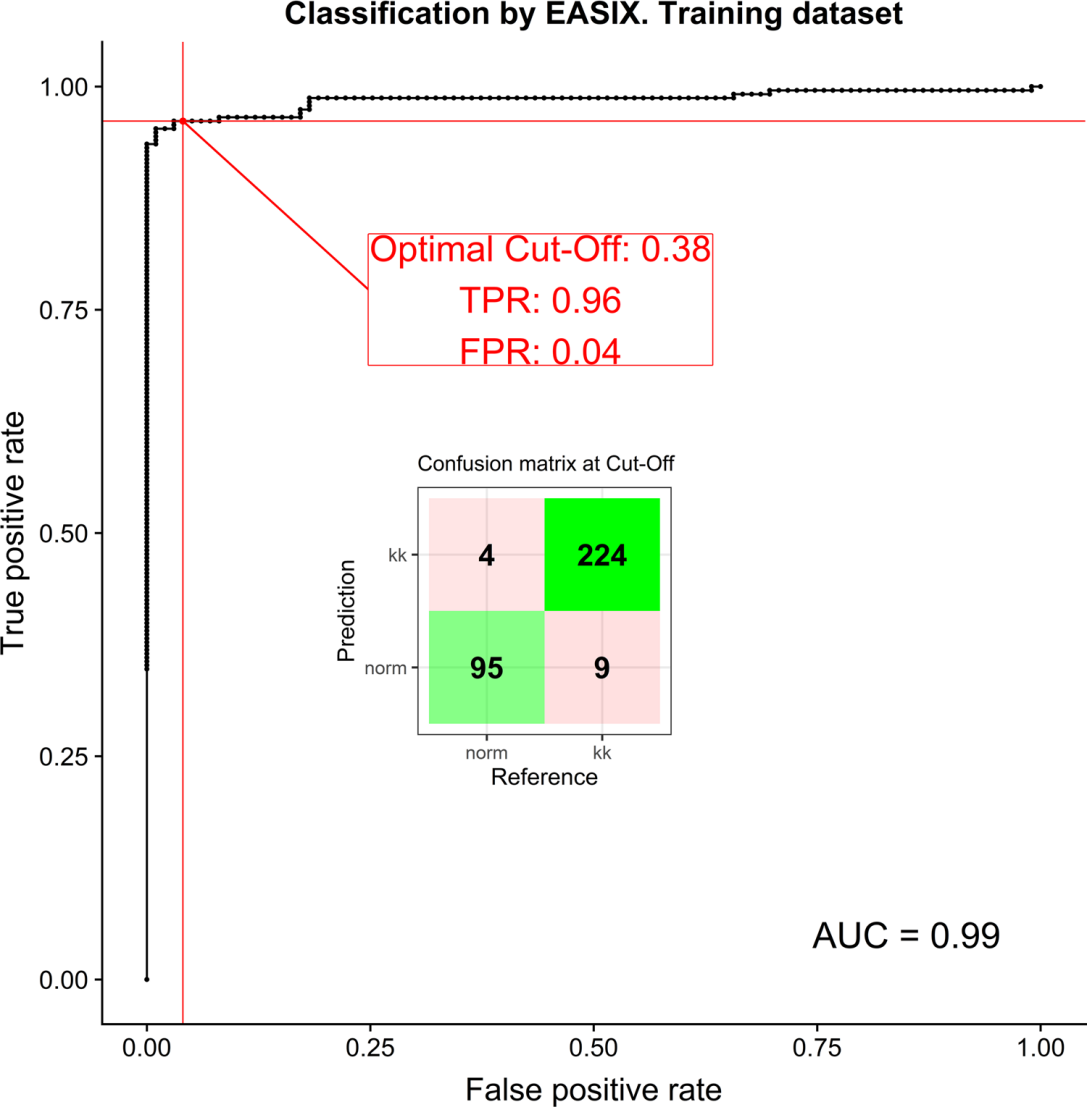
Classification by EASIX. Training dataset

**Fig. 3:**
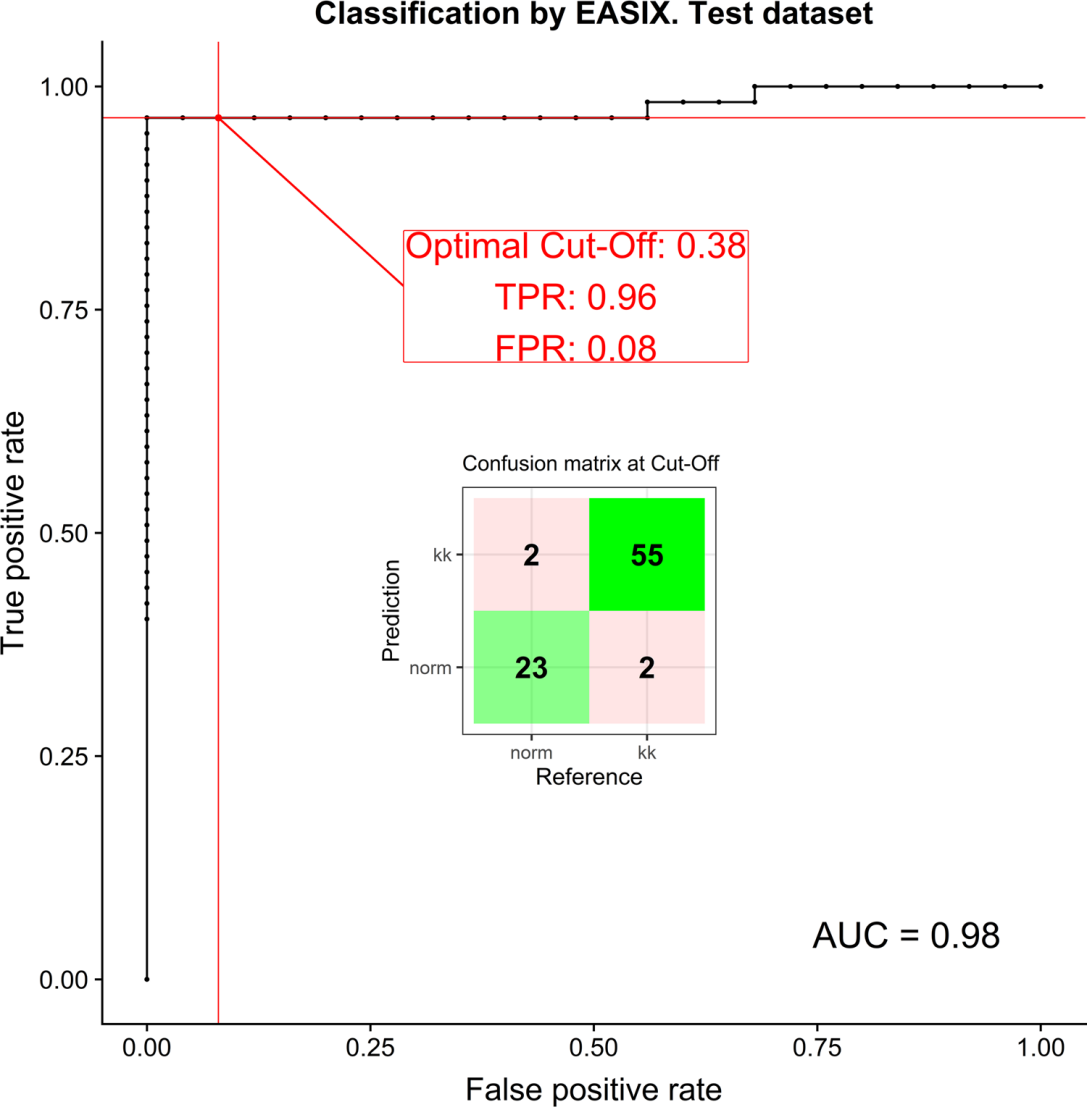
Classification by EASIX. Test dataset

The logits from the final model were transformed to probabilities that were further analyzed via ROC to derive the optimal cutoff (Figs. 2 and 3). Finally, the logistic model and the estimated cutoff point were applied to the test data set.

The resulting probabilities were analyzed further with ROC method to derive the optimal cutoff point.

## Results

A total of 414 eyes were analyzed with the Pentacam and divided into the two groups: 124 subjects with bilateral normal corneas evaluated for refractive surgery and 290 with keratoconus.

Table 1 presents the between-eye asymmetry, range, and SDs of all the indices analyzed in subjects with bilateral normal eyes and in patients with keratoconus.

**Table 1:** Between-eye asymmetry, range, and SDs in all the indices analyzed in subjects with bilateral normal eyes and in patients with keratoconus

	Normal (*n* = 124)		Keratokonus (*n* = 290)		
Variable	Range	Median (Q1/Q3)	Range	Median (Q1/Q75)	*P*-value^a^
ART Avg.:	1.00 to 274.00	44.00 (19.00/81.50)	0.00 to 666.00	114.50 (40.25/196.00)	< 0.001
ART Max.:	1.00 to 235.00	51.50 (19.75/92.25)	0.00 to 526.00	78.50 (26.25/145.50)	< 0.001
ART Min.:	1.00 to 956.00	141.50 (68.00/240.50)	0.00 to 1552.00	164.00 (65.00/319.75)	0.12
Asph. Q Back (30°):	0.00 to 0.51	0.12 (0.05/0.20)	0.01 to 9999.29	0.48 (0.21/0.85)	< 0.001
Asph. Q Front (30°):	0.00 to 0.33	0.04 (0.02/0.07)	0.00 to 2.69	0.33 (0.15/0.67)	< 0.001
Astig B (D):	0.00 to 0.80	0.10 (0.07/0.20)	0.00 to 3.60	0.40 (0.20/0.70)	< 0.001
Astig F (D):	0.00 to 4.80	0.40 (0.20/0.70)	0.00 to 24.90	1.65 (0.70/3.00)	< 0.001
Axis B (flat):	0.60 to 178.20	157.85 (15.60/166.70)	0.40 to 175.30	112.05 (75.62/140.57)	< 0.001
Axis F (flat):	0.10 to 175.60	125.70 (13.22/156.03)	0.70 to 176.70	98.30 (54.12/129.78)	0.131
BAD D:	0.00 to 1.20	0.28 (0.13/0.53)	0.00 to 37.96	3.89 (1.92/7.26)	< 0.001
BAD Daa:	0.01 to 2.06	0.34 (0.14/0.62)	0.00 to 5.01	0.86 (0.30/1.47)	< 0.001
BAD Dam:	0.01 to 2.15	0.47 (0.18/0.84)	0.00 to 4.80	0.72 (0.24/1.34)	< 0.001
BAD Db:	0.01 to 1.91	0.41 (0.22/0.95)	0.02 to 48.08	4.70 (2.11/8.57)	< 0.001
BAD De:	0.00 to 2.16	0.52 (0.24/0.87)	0.00 to 60.16	5.53 (2.72/9.49)	< 0.001
BAD Df:	0.00 to 2.50	0.52 (0.16/0.94)	0.09 to 41.99	5.38 (2.77/10.07)	< 0.001
BAD Dk:	0.00 to 1.67	0.19 (0.09/0.45)	0.01 to 25.55	3.18 (1.58/5.91)	< 0.001
BAD Dp:	0.00 to 3.50	0.44 (0.16/0.79)	0.02 to 134.79	4.04 (1.62/8.27)	< 0.001
BAD Dr:	0.01 to 3.34	0.42 (0.19/0.76)	0.01 to 27.47	2.35 (1.06/4.44)	< 0.001
BAD Dt:	0.01 to 1.47	0.20 (0.10/0.36)	0.02 to 23.13	1.01 (0.53/1.93)	< 0.001
BAD Dy:	0.01 to 3.34	0.59 (0.28/1.23)	0.00 to 15.20	0.85 (0.40/1.55)	0.024
BFS Back 8 mm:	0.00 to 0.18	0.04 (0.02/0.07)	0.00 to 1.96	0.23 (0.10/0.42)	< 0.001
BFS Front 8 mm:	0.00 to 0.14	0.03 (0.02/0.05)	0.00 to 2.25	0.25 (0.12/0.48)	< 0.001
CKI:	0.00 to 0.02	0.00 (0.00/0.01)	0.00 to 0.31	0.03 (0.01/0.07)	< 0.001
Ele B BFS 8 mm Apex:	0.00 to 6.00	1.00 (1.00/2.25)	0.00 to 133.00	15.00 (7.00/27.00)	< 0.001
Ele B BFS 8 mm Max. 4 mm Zone:	0.00 to 24.00	3.00 (1.00/5.00)	0.00 to 186.00	25.00 (12.00/43.00)	< 0.001
Ele B BFS 8 mm Thinnest:	0.00 to 10.00	2.00 (1.00/4.00)	0.00 to 280.00	26.00 (12.00/44.00)	< 0.001
Ele F BFS 8 mm Apex:	0.00 to 4.00	0.00 (0.00/1.00)	0.00 to 75.00	6.00 (2.00/12.00)	< 0.001
Ele F BFS 8 mm Max. 4 mm Zone:	0.00 to 15.00	1.00 (0.00/2.00)	0.00 to 66.00	13.00 (6.00/21.00)	< 0.001
Ele F BFS 8 mm Thinnest:	0.00 to 4.00	1.00 (0.00/2.00)	0.00 to 233.00	11.00 (6.00/20.00)	< 0.001
Enh. BFS Back 8 mm:	0.00 to 0.19	0.04 (0.02/0.07)	0.00 to 1.93	0.13 (0.06/0.29)	< 0.001
Enh. BFS Front 8 mm:	0.00 to 0.12	0.04 (0.02/0.06)	0.00 to 2.08	0.18 (0.08/0.35)	< 0.001
IHA:	0.10 to 16.50	2.10 (1.07/3.62)	0.00 to 849.70	13.20 (5.42/24.57)	< 0.001
IHD:	0.00 to 0.02	0.00 (0.00/0.00)	0.00 to 0.68	0.04 (0.02/0.06)	< 0.001
ISV:	0.00 to 34.00	2.00 (1.00/4.25)	0.00 to 195.00	34.50 (16.00/60.00)	< 0.001
IVA:	0.00 to 0.18	0.03 (0.02/0.05)	0.00 to 2.24	0.34 (0.14/0.65)	< 0.001
K1 B (D):	0.00 to 0.50	0.10 (0.00/0.10)	0.00 to 5.30	0.40 (0.20/1.00)	< 0.001
K1 (D):	0.00 to 2.10	0.30 (0.10/0.50)	0.00 to 26.90	2.05 (0.70/4.70)	< 0.001
K2 B (D):	0.00 to 0.50	0.10 (0.00/0.10)	0.00 to 5.80	0.70 (0.30/1.30)	< 0.001
K2 (D):	0.00 to 2.70	0.30 (0.10/0.60)	0.10 to 28.70	3.45 (1.40/5.90)	< 0.001
KI:	0.00 to 0.05	0.01 (0.01/0.02)	0.00 to 0.83	0.10 (0.05/0.19)	< 0.001
K Max Front (D):	0.00 to 2.70	0.30 (0.17/0.70)	0.00 to 41.10	5.15 (2.53/9.45)	< 0.001
K Max Front X:	0.00 to 3.13	0.34 (0.14/0.75)	0.00 to 3.84	0.27 (0.11/0.53)	0.037
K Max Front Y:	0.00 to 6.07	0.71 (0.27/1.64)	0.00 to 7.25	0.60 (0.24/1.14)	0.124
Kmaxmag	0.00 to 4.14	0.45 (0.21/0.88)	0.00 to 3.80	0.60 (0.25/1.03)	0.087
Pachminmag	0.00 to 0.68	0.15 (0.07/0.26)	0.00 to 4.02	0.22 (0.11/0.35)	< 0.001
Pachy Apex:	0.00 to 39.00	7.00 (3.00/13.00)	0.00 to 315.00	26.00 (12.00/42.00)	< 0.001
Pachy Min.:	0.00 to 47.00	7.00 (3.00/13.00)	1.00 to 279.00	29.00 (14.00/51.75)	< 0.001
Pachy Min X:	0.00 to 0.86	0.20 (0.08/0.36)	0.00 to 3.88	0.19 (0.10/0.32)	0.863
Pachy Min Y:	0.00 to 0.87	0.15 (0.07/0.32)	0.00 to 3.96	0.22 (0.10/0.41)	0.025
Rm B (mm):	0.00 to 0.27	0.06 (0.03/0.10)	0.00 to 2.70	0.47 (0.22/0.88)	< 0.001
Rm F (mm):	0.00 to 0.24	0.03 (0.02/0.06)	0.00 to 2.77	0.40 (0.17/0.75)	< 0.001
R Min (mm)	0.00 to 0.45	0.05 (0.02/0.12)	0.00 to 2.92	0.65 (0.31/1.21)	< 0.001
RPI Avg.:	0.00 to 0.52	0.07 (0.03/0.12)	0.01 to 19.93	0.60 (0.24/1.23)	< 0.001
RPI Max.:	0.00 to 0.53	0.12 (0.05/0.21)	0.00 to 229.08	0.88 (0.34/1.93)	< 0.001
RPI Max. Axis:	0.00 to 284.90	82.55 (21.58/173.50)	0.00 to 288.70	33.70 (15.00/93.78)	< 0.001
RPI Min.:	0.00 to 0.53	0.09 (0.05/0.17)	0.00 to 353.38	0.60 (0.27/1.08)	< 0.001
RPI Min. Axis:	0.00 to 326.20	153.80 (122.77/177.15)	0.00 to 345.00	116.20 (38.42/157.47)	< 0.001

*Q1* first quartile, *Q3* third quartile, *ICC* intraclass correlation

Median absolute differences within two groups were all significantly different from 0, Wilcoxon test *p* < 0.05

^a^Non-parametric Mann–Whitney test

Furthermore Table 1 presents the mean value for each variable of each eye analyzed in both groups.

Table 2 shows the sensitivities and specificities of the intereye asymmetry value for each variable.

**Table 2:** ROC analysis of intereye correlation to discriminate normal versus keratoconus

Variable	Cutoff^a^	AUC	Sensitivity	Specificity	Accuracy
IHD:	0.01	0.97	0.92	0.9	0.92
BAD Df:	1.36	0.95	0.88	0.88	0.88
K Max Front (D):	1	0.94	0.89	0.88	0.89
BAD Dk:	0.62	0.94	0.89	0.89	0.89
Ele F BFS 8 mm Thinnest:	3	0.94	0.89	0.9	0.89
Ele B BFS 8 mm Apex:	4	0.94	0.87	0.85	0.86
BAD D:	0.78	0.94	0.88	0.88	0.88
IVA:	0.08	0.94	0.88	0.88	0.88
Rm F (mm):	0.09	0.93	0.87	0.85	0.86
BAD Db:	1.13	0.93	0.86	0.85	0.86
K2 (D):	0.8	0.93	0.87	0.87	0.87
ISV:	7	0.93	0.86	0.85	0.85
KI:	0.03	0.92	0.86	0.89	0.86
R Min (mm)	0.15	0.92	0.85	0.85	0.85
Ele B BFS 8 mm Thinnest:	6	0.92	0.86	0.83	0.85
BAD De:	1.27	0.92	0.85	0.85	0.85
Ele F BFS 8 mm Apex:	2	0.92	0.81	0.94	0.85
BFS Front 8 mm:	0.07	0.92	0.85	0.83	0.85
Ele B BFS 8 mm Max. 4 mm Zone:	7	0.92	0.86	0.84	0.86
BAD Dp:	1.02	0.91	0.84	0.85	0.85
RPI Avg.:	0.15	0.91	0.84	0.84	0.84
K2 B (D):	0.2	0.91	0.86	0.81	0.84
Ele F BFS 8 mm Max. 4 mm Zone:	4	0.91	0.85	0.89	0.86
Rm B (mm):	0.14	0.9	0.82	0.82	0.82
Asph. Q Front (30°):	0.11	0.89	0.83	0.83	0.83
BFS Back 8 mm:	0.09	0.89	0.82	0.86	0.83
RPI Max.:	0.26	0.89	0.82	0.83	0.82
CKI:	0.02	0.89	0.72	0.96	0.79
BAD Dt:	0.41	0.89	0.81	0.81	0.81
RPI Min.:	0.2	0.89	0.83	0.82	0.83
Enh. BFS Front 8 mm:	0.07	0.88	0.79	0.8	0.79
K1 B (D):	0.2	0.88	0.8	0.85	0.81
K1 (D):	0.6	0.88	0.81	0.83	0.81
BAD Dr:	0.84	0.87	0.79	0.78	0.79
IHA:	4.2	0.86	0.79	0.79	0.79
Pachy Min.:	14	0.84	0.75	0.76	0.75
Asph. Q Back (30°):	0.2	0.84	0.75	0.74	0.75
Astig B (D):	0.2	0.83	0.78	0.73	0.77
Pachy Apex:	13	0.82	0.74	0.73	0.73
Astig F (D):	0.7	0.82	0.77	0.78	0.77
Enh. BFS Back 8 mm:	0.07	0.8	0.72	0.73	0.72
BAD Daa:	0.5	0.71	0.66	0.65	0.65
ART Avg.:	66	0.71	0.66	0.65	0.66
Pachminmag	0.18	0.61	0.56	0.56	0.56
BAD Dam:	0.55	0.61	0.59	0.59	0.59
ART Max.:	61	0.61	0.59	0.59	0.59
BAD Dy:	0.69	0.57	0.57	0.56	0.57
Pachy Min Y:	0.18	0.57	0.57	0.56	0.57
Kmaxmag	0.52	0.55	0.54	0.54	0.54
ART Min.:	152	0.55	0.53	0.53	0.53
Pachy Min X:	0.2	0.49	0.48	0.48	0.48
Axis F (flat):	107.3	0.45	0.45	0.45	0.45
K Max Front Y:	0.65	0.45	0.47	0.48	0.47
K Max Front X:	0.3	0.44	0.46	0.46	0.46
RPI Max. Axis:	48.8	0.38	0.39	0.39	0.39
Axis B (flat):	130.6	0.35	0.34	0.34	0.34
RPI Min. Axis:	142.5	0.33	0.32	0.32	0.32

*AUC* area under the ROC curve, *ROC* receiver operating characteristics

^a^The point where the absolute difference between sensitivity and specificity is minimized

The mean between-eye differences were statistically significant for almost all the variables when comparing the normal eyes with the keratoconic eyes (Mann–Whitney test, *P* < 0.05) except for Axis F (flat), Art min, K Max Front, Pachy min x, and k_max_mag.

An intereye asymmetry in the posterior elevation of 8 mm had 86% sensitivity and 84% specificity, discriminating normal subjects from keratoconic patients. In normal eyes, the mean intraclass correlation coefficients for central corneal thickness (CCT), pachymetry at the thinnest point (TP), and posterior elevation at the thinnest point of the cornea (PETP) were 0.74, 0.75, and 0.86, respectively; in keratoconic eyes, the mean intraclass correlation coefficients for CCT, TP, and PETP were 0.73, 0.76, and 0.83, respectively.

The point that minimizes the absolute difference between sensitivity and specificity is given by 0.38. The resulting sensitivity and specificity are 0.961 and 0.96, respectively.

The coefficients were used to calculate probabilities for the test data set. The cutoff 0.38 was used for class prediction. The result for the test data set is shown in Fig. 2.

The resulting sensitivity and specificity for test data set are 0.965 and 0.92, respectively.

## Discussion

The present study was set to explore the discriminative capacity of intraindividual topographic and tomographic intereye asymmetry parameters in differentiating between healthy subjects and keratoconus patients.

Measurements on both eyes of a person are often correlated. This means that measurements on one eye are more similar to those of the other eye than measurements on an unrelated person [1, 12]. On the other hand asymmetry between keratoconic eyes has been reported previously [6–8, 13] and the Rabinowitz and MacDonnell criteria for keratoconus published 20 years ago already included a difference between the right and left central corneal power [2, 3]. Recent studies [11, 14, 15] reveal a renewed interest in the aspect of intereye asymmetry.

We found statistically significant increased intereye difference in posterior elevation and pachymetry values (except Pachy_Min_X) in keratoconus patients compared to normal subjects, confirming previous reports [11, 14]. In addition, we observed significantly different BFS front, RMS (cornea) parameters in keratoconus compared to healthy individuals. The finding in one eye predicts the finding in the fellow eye almost perfectly in healthy persons and moderately in keratoconus patients. The decreased correlation between values measured in the two eyes of the same subject with keratoconus is a consequence of the asymmetrical nature of this disease.

The detailed analysis of the discriminative capacity based on intereye asymmetry is the subject of previous studies [7, 8, 14, 16]. However only the most recent study is based on modern Scheimpflug technology including topographic and tomographic parameters, for example, pachy min galletti cutoff.

Henriquez et al. demonstrated an intereye asymmetry of 0.75 D in steep keratometry with an AUROC value of 0.92 (86% sensitivity and 90% specificity, discriminating normal subjects from keratoconic patients) confirming the postulation of Rabinowitz from 1995 [2, 14]. Our retrospective analysis revealed a cutoff value of 0.6 D for steep keratometry (K1) with an AUROC value of 0.88 (81% sensitivity and 83% specificity).

Interestingly, Henriquez et al. found that the intereye asymmetry at the posterior elevation (PETP) had a similar AUROC value (0.91), with 85% sensitivity and 88% specificity [14]. Few other reports document asymmetry in keratoconic eyes [11, 15]. Zadnik et al. reported mean differences between keratoconic eyes: spherical equivalent, 3.00 D; cylinder power, 1.50 D; and corneal curvature, 3.5 to 4.0 D [8]. Today, the use of corneal pachymetry maps and posterior elevation maps is almost mandatory for evaluating refractive surgery candidates and keratoconic patients. Researchers have reported statistically significant differences between normal eyes and keratoconic eyes with respect to parameters such as CCT, TP, PETP, distance, and volume.

Some suggest using these parameters to distinguish between normal eyes and keratoconic eyes. It is evident from our data that the intereye asymmetry was greater among keratoconic patients than between eyes with normal corneas, in parameters derived from Scheimpflug imaging. The mean intereye asymmetry was statistically significant for nearly all the variables analyzed when comparing the normal subjects with the keratoconic patients. The median intereye asymmetry in the normal group in pachymetry at the apex of the cornea was 7 μm (range from 3.00 to 13 μm), and median at the TP was also 7 μm, in accordance with those reported by Khachikian et al. who reported 8.8 and 9.0 μm, respectively [17]. Falavarjani et al. reported an intereye asymmetry of 8.42 μm (range: 0 to 30) at the TP for normal subjects [18]. In contrast, our results show that the median intereye asymmetry in the keratoconus group at the apex of the cornea was 26.00 μm and at the TP 29.00 μm.

Based on our data, a greater than 26.06-μm difference in the apical thickness between eyes represents 3.2% of the normal population and 47.9% of the keratoconic population. A greater than 27.48-μm difference in the TP between eyes represents 3.2% of the normal population and 52.4% of the keratoconic population. Our results showed that an intereye asymmetry of 13.00 μm in the CCT had 74% sensitivity and 73% specificity, discriminating normal subjects from keratoconic patients. When evaluating posterior elevation, the mean intereye asymmetry in the normal group at the posterior corneal elevation was 3.80 μm in accordance with 3.62 μm reported by Falavarjani et al. at the maximum posterior elevation.

Saad et al. found a mean intereye central corneal thickness asymmetry in the normal group of 6.0 ± 5.0 μm at the thinnest corneal thickness. None of the patients in the normal group had an intereye central corneal thickness difference greater than 18 μm [16]. In 2008, Khachikian et al. found a thinnest intrasubject corneal thickness difference of 9.00 ± 8.3 μm using a Scheimpflug system [17]. With the same equipment, Falavarjani et al. showed similar results, with a difference of 8.42 μm [18]. Henriquez et al. found a between-eye central corneal thickness difference equal to 10.28 ± 7.89 μm [14]. Our results were slightly lower than those previously reported in the literature: this might be explained by the fact that the normal group was composed of LASIK candidates, for whom a subjective appreciation of the degree of corneal enantiomorphism may influence the selection process.

The sensitivity and specificity of the intereye central corneal thickness difference for the discrimination between the normal and keratoconic group were 74% and 73%, respectively, with 13 μm as a cutoff. Other individual right and left eye differences yield variable sensitivity and specificity. The Eye Asymmetry Index (EASIX) had in training dataset 0.99 and test dataset 0.98 area under the curve.

A study of Módis et al. showed that that the slit-scanning technology is very well suited for the examination and diagnosis of keratoconus [19]. When corneal opacities are present, the slit-scanning technology is not precise anymore, but our group of patients with keratoconus was only composed of clear corneas.

Our data provide further evidence that keratoconus is markedly asymmetric between the eyes, both in an absolute sense and when compared with normal, healthy subjects.

A discriminant function constructed from intereye difference of three corneal indices may be accurate and useful for the topography-based detection of advanced keratoconus.

To avoid postoperative LASIK ectasia, one important task for the refractive surgeon is to detect early forms of subclinical keratoconus. In this study, we aimed to define the normal tolerable range of asymmetry between the right and left eyes using the combined elevation and placido topography. In the future, incorporating such data in an automated artificial intelligence may improve the detection ability.

The limitations of our study include the retrospective design and no further staging or classification of the keratoconus group. We further relied on only one high-quality measurement per eye to calculate corneal asymmetry.

The elevation data obtained by the Pentacam do not necessarily match data obtained by other diagnostic devices [20] and therefore the reported diagnostic models might only apply to the Pentacam.

But from our point of view, the use of the Pentacam was indicated due to the reliable measurement of the corneal curvature variables. In studies by Shajari et al. it has also been proven that the Pentacam is a reliable instrument for measurement of corneal curvature variables [9].

Therefore, we suggest validating the diagnostic value of corneal asymmetry with other Scheimpflug, Placido, Hybrid, or OCT tomography devices.

To further improve the diagnostic potential of single-asymmetry descriptors, a multivariate Eye Asymmetry Index (EASIX) was developed following discriminant analysis of Scheimpflug-derived parameters. These parameters were preselected according to their single discriminant capacity between normal and keratoconic asymmetry.

In addition to established isolated single-side parameters the new Eye Asymmetry Index (EASIX) takes advantage of the high anatomical symmetry between the paired organ visual system.

Future longitudinal studies should be initiated to test the value of EASIX in serving as an early screening and a keratoconus progression marker.
